# Disruption of Copper Redox Balance and Dysfunction under *In Vivo* and *In Vitro* Alzheimer’s Disease Models

**DOI:** 10.1021/envhealth.4c00175

**Published:** 2024-11-13

**Authors:** Yiteng Xia, Karl W. K. Tsim, Wen-Xiong Wang

**Affiliations:** † School of Energy and Environment and State Key Laboratory of Marine Pollution, City University of Hong Kong, Kowloon, Hong Kong, China; ‡ Research Centre for the Oceans and Human Health, City University of Hong Kong Shenzhen Research Institute, Shenzhen 518057, China; § Division of Life Science, Hong Kong University of Science and Technology, Clear Water Bay, Kowloon, Hong Kong, China

**Keywords:** Alzheimer’s disease, β-amyloid, Cu homeostasis, Cu valence transformation, Diagnosis

## Abstract

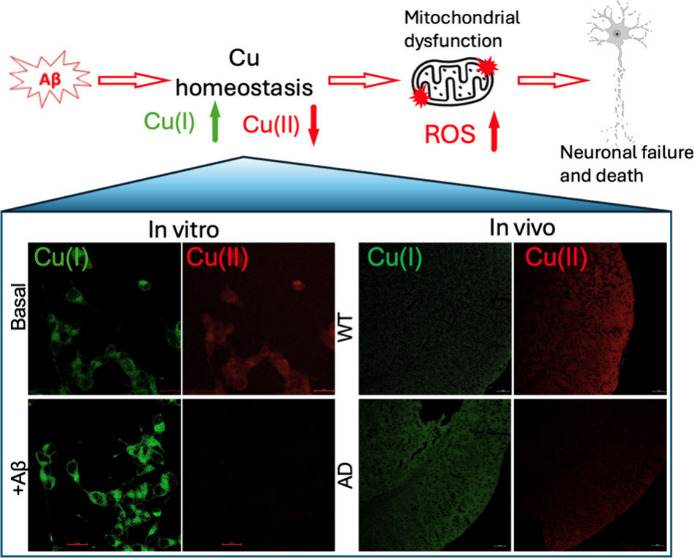

Alzheimer’s disease (AD) is a neurodegenerative disorder disease mainly caused by extracellular senile plaques (SP) formed by β-amyloid (Aβ1–42) protein deposits. Copper (Cu) is an essential metal involved in neural system, and its homeostasis is the key to maintain its proper function. Herein, the subcellular locations of Cu(I) and Cu(II) in human neurodegenerative disease SH-SY5Y cells and AD mouse brains were imaged. We found that the content of Cu(II) decreased while that of Cu(I) increased under Aβ exposure, which were further verified in the brain tissues of the AD mouse model, strongly suggesting the disruption of Cu homeostasis under Aβ exposure or AD. Remarkably, the mitochondrial and lysosomal Cu(II) decreased significantly, whereas Cu(I) decreased in mitochondria but increased in lysosome. Lysosomes digested the damaged mitochondria via mitophagy to remove excess Cu(I) and maintain Cu homeostasis. The Aβ induced Cu(I) in mitochondria resulted in an overformation of reactive oxygen species and altered the morphology of this organelle. Due to the oxidative stress, glutathione (GSH) was converted into glutathione disulfide (GSSG), and Cu(I) bound with GSH was further released into the cytoplasm and absorbed by the lysosome. Transcriptomic analysis showed that genes (ATP7A/B) related to Cu transportation were upregulated, whereas genes related to mitochondrial complex were down-regulated, representing the damage of this organelle. This study demonstrated that Aβ exposure caused the disruption of intracellular homeostasis by reducing Cu(II) to Cu(I) and damaging the mitochondria, which further triggered detoxification by the lysosome. Our finding provided new insights in Aβ and AD induced Cu redox transformation and toxicity.

## Introduction

Alzheimer’s disease (AD) is a neurodegenerative disorder characterized by cognitive and behavioral impairment that significantly interferes with social and occupational functioning.^1^ This disease affects over 55.0 million people worldwide and is ranked as the fifth leading cause of death globally.^2^ Several hypotheses have been put forward to postulate the pathogenesis of AD, including oxidant stress^3,4^ and mutations of gene involved in AD occurrence and development.^5−7^ The current therapeutics for AD can relieve the symptoms but cannot reverse neuronal and synaptic dysfunction in patients.

Pathological changes of AD are characterized by extracellular senile plaques (SP) formed by β-amyloid (Aβ1–42) protein deposits,^2^ neurofibrillary tangles in neuronal cells formed by hyperphosphorylation of tau,^8^ and neuronal or glial deficiency caused by neuroinflammation.^9^ The amyloid cascade hypothesis served as the dominant framework for AD studies.^10^ The existence of amyloid plaques in the patient’s brain is considered as the primary hallmark of AD.^11^ Aβ is generated from the amyloid precursor protein^12^ by sequential cleavage of β- and γ-secretase.^7^ After secretion, Aβ first aggregates into different soluble species and then changes its conformation into cross-β-sheet fibrils to form plaques. Amyloid fibrils are insoluble proteinaceous materials in a wide range of protein-misfolding diseases, including Alzheimer’s and prion diseases as well as several types of systemic amyloidosis.^13^ Aβ aggregates can directly interact with the lipid and cholesterol components of the cell membrane. It can destroy membrane integrity and permeability, causing excessive Ca influx and leading to long-term potentiation (LTP) inhibition and neuronal death.^14^ By changing the morphology and density of synapses, Aβ oligomers led to the impairment of synaptic plasticity.^15^ Aβ could interact with tau proteins to exert toxic tauopathy (a class of neurodegenerative diseases characterized by the aggregation of abnormal tau protein),^16^ and contribute to other AD pathological features including neuroinflammation,^17^ oxidative stress, and mitochondrial dysfunction,^18^ with subsequent neuronal death and dysfunction.

Recent studies indicated that many metals including Cu, Fe, Zn, and Mn contributed to neurodegeneration and AD development.^19^ Cu is an essential metal with two different oxidation states [Cu(II) and Cu(I)],^13^ which can be transformed with each other in cells. This element is critical for various processes including cellular respiration,^20^ apoptosis,^21^ and intracellular Fe metabolism.^22^ Cu can catalyze the formation of free radical species, e.g., reactive oxygen species,^18,23^ and excess Cu results in overproduction of ROS which affects the protein oxidation and cleavage of DNA and RNA.^24^

In the neuro system, Cu contributes to neurotransmitter synthesis,^25^ epigenetics, and construction of the extracellular matrix.^26^ Cu modulates the function of several receptors, including γ-aminobutyric acid type A (GABAA) receptors,^27^*N*-methyl-d-aspartate (NMDA) receptors,^28^ and voltage-gated Ca^2+^ channels. In addition, amyloid precursor protein,^29^ prion protein,^30^ and AMP-activated protein kinase are also altered by Cu.^27^ Many neurological disorders and diseases such as Menkes disease,^31^ Wilson’s disease,^20^ motor neuron disease,^32^ and Alzheimer’s disease^33^ are also related to disturbance of Cu homeostasis.

Aβ may interact with Cu ion, especially Cu(II), including the formation Cu-stabilized oligomeric Aβ species.^34^ The Aβ-Cu complex in the brain contributes to peptide toxicity by the production of radicals and hydrogen peroxide and peptide aggregation.^35^ The Aβ-Cu complex catalyzes the activation of O_2_ into superoxide anion^36^ and leads to oxidative stress. In aquatic solution, Aβ could alter the Cu valence by reducing Cu(II) to Cu(I) with Aβ addition.^37,38^ However, Aβ-induced transportation or transformation of Cu ions *in vivo* or *in vitro* remains essentially unknown. With the recent development of fluorescent probes, the location and valence changes of Cu(I) and Cu(II) in cells can be visualized. In the present study, CF4 (a Cu^+^-specific fluorescence prob)^39^ and CD649.2 (a Cu^2^+-specific fluorescence probe)^40^ were employed to visualize the location and transformation of Cu in cell and tissue sections. These two probes showed excellent biocompatibility with high signal-to-noise ratio^41^ and were used to visualize the changes in Cu valences and location in cells under Aβ exposure. The human neuroblastoma cell line SH-SY5Y has been frequently used as an in vitro model for neurodegenerative disease studies including AD.^1,2,42,43^ Earlier, the addition of Aβ in SH-SY5Y culture medium was used as an in vitro model in parallel with the mouse AD model.^44^ The 5XFAD mouse was widely applied as an in vivo model in AD research, and the mutated mice were found to accumulate Aβ in the hippocampus and cortex.^45^ Previous studies used both SH-SY5Y cells and 5XFAD mice as in vitro and in vivo AD models.^46,47^ In this study, we hypothesized that Aβ addition reduces intracellular Cu(II) to Cu(I), and excessive Cu(I) might lead to cellular damage and death afterward.

## Materials and Methods

### In Vitro Cell Model

The human SH-SY5Y cell line was widely applied as an *in vitro* model for different neurodegenerative diseases including AD,^48^ and the Aβ toxicity on this cell line was studied earlier.^12^ Dulbecco’s modified Eagle’s medium (DMEM) supplemented with 100 IU/mL of penicillin, 100 μg/mL of streptomycin, and 15% fetal bovine serum was applied as the full growth medium for SH-SY5Y cells. The cells were incubated at 37 °C in water saturated with 5% CO_2_. After reaching 70% confluence, SHSY5Y cells were harvested with 1 mL of 0.25% trypsin for 3 min and trypsinization was stopped by adding 10 mL full growth medium.

To prepare the aggregated beta-amyloid (Aβ1–42) fibrils, purified synthetic beta-amyloid (Aβ1–42) (GL Biochem, Shanghai, China) was first mixed with hexafluoroisopropanol and sonicated for 20 min at room temperature. Then the Aβ1–42 solution was dried overnight to prepare the Aβ1–42 peptide film. After that, 0.5 mg of Aβ1–42 peptide film was resuspended with 20 μL of DMSO and 1120 μL of 10 mmol/L HCl. Then, the Aβ1–42 solution was vortexed for 1 min and incubated at 37 °C for 6 days to result in an Aβ1–42 fibrils mixture. The final concentration of Aβ1–42 fibrils was 100 μmol/L. The Aβ1–42 peptides or fibrils were freeze-dried and coated with gold, and their morphology was observed via scanning electron microscopy (SEM) (Carl Zeiss).

### Thioflavin T Fluorescence Assays and Cytotoxicity Study

Thioflavin T (ThT) fluorescence dye was applied for the determination of Aβ1–42 fibrils. Upon ThT binding to amyloid fibrils, the central C–C bond connecting the benzothiazole and aniline rings immobilized rotationally and gave a strong fluorescence signal.^49^ ThT solution was mixed with Aβ solution at a final concentration of 20 μmol/L. The intensity of ThT fluorescence was measured with λex = 435/λem = 488 nm with a microplate reader (FlexStation Multimode Microplate Reader).

The cytotoxicity of Aβ was determined by via MTT assay. In brief, SH-SY5Y cells were seeded in a 96-well plate at a density of 20000 cells/well and cultured for 12 h. After that, various concentrations (1–10 μmol/L) of aggregated or nonaggregated Aβ solution were mixed with the cell culture medium and cultured for 12 and 24 h separately. After treatment, 3-(4,5-dimethylthiazol-2-yl)-2,5-diphenyltetrazolium bromide (MTT) solution was added at a final concentration of 0.5 mg/mL. After 2 h of incubation, DMSO was used to dissolve the produced purple crystal. Absorbance was measured by a microplate reader (FlexStation Multimode Microplate Reader) at 570 nm.

### In Vivo Mouse Model

The 5xFAD mice were chosen and created from wild-type C57BL/6 mice as the *in vivo* model. 5xFAD mice express human APP and PSEN1 transgenes with a total of five AD-linked mutations: the Swedish (K670N/M671L), Florida (I716 V), and London (V717I) mutations in APP, and the M146L and L286V mutations in PSEN1. 5XFAD mice exhibited amyloid deposition, gliosis, and progressive neuronal loss accompanied by cognitive and motor deficiencies, recapitulating many of the features of human AD. Both male and female littermate mice aged 12 months old were used in this study, which reflected the late stage of AD.

The wild-type C57BL/6 mice were obtained from the Animal and Plant Care Facility of Hong Kong University of Science and Technology (HKUST), and the 5xFAD mice were purchased from Shanghai Model Organisms Center (Shanghai, China) and cared for according to the guidelines of Department of Health, The Government of Hong Kong SAR. The experimental procedures were approved by the Animal Ethics Committee at the University (Reference No.: (15–50) in DH/SHS/8/2/2 Pt.2). Up to six mice were housed per cage, and the colony room was kept on a 12:12 L:D schedule with the lights on from 7:00 am to 7:00 pm daily. Experimental mice were euthanized by CO_2_. Intact brains were fixed with 4% paraformaldehyde overnight at 4 °C and then perfused with a sucrose solution. Afterward, the brain tissues were embedded in OCT and frozen at −80 °C overnight and then sectioned coronally into 20 μm thickness using Thermo CryoStar NX 70 Cryostat (Thermo Fisher Scientific) (Figure S1). The section was stored at −20 °C for the following experiments. The fixed brain tissue sections were incubated with PBST for 10 min for rehydration. Then sections were blocked with 5% BSA/PBST at room temperature for 1 h. Afterward, the brain sections were incubated with primary antibody, anti-Aβ (Millipore, MA) at 4 °C overnight followed by labeling with Alexa Fluor 488 or Alexa Fluor 647-conjugated anti-rabbit antibodies (1:200 V/V) for 2 h at room temperature followed by mounting. The Aβ in brain tissue section was observed with the excitation and emissions wavelengths at 488 nm and 520–550 or 647 nm and 670–700 nm for fluorescent signal capture.

For Cu visualization, the tissue sections were stained with the Cu(I) specific probe CF4 and the Cu(II) specific probe CD649.2. Here, tissue sections before mounting were stained with the CF4 probe at a final concentration of 50 μmol/L or CD649.2 at a final concentration of 100 μmol/L for 1 h. The excitation and emissions wavelengths were set at 488 nm and 530–590 nm for Cu(I) fluorescent signal capture. The CD649.2 probe was excited at 647 nm, and emissions between 650 and 700 nm were collected as Cu(II)-specific signals. The confocal images were captured by a confocal microscope LSM900 equipped with Airyscan in channel mode (Carl Zeiss). The mean fluorescence intensity (MFI) of Cu^+^ and Cu^2+^ were quantified based on the confocal images.

### Immunofluorescence

For immunofluorescent analysis, about 1 × 10^5^ SH-SY5Y cells were seeded in a confocal dish (NEST Biotechnology, Wuxi, China) and cultured for 24 h. The cells were then pretreated with 1 or 5 μmol/L Aβ1–42 fibrils or nonaggregated Aβ for another 24 h with FBS free culture medium. After culturing, cells were washed three times with 1X PBS to discard the residual Aβ.

The cellular Cu was probed by a Cu(I) specific probe CF4 and a Cu(II) specific probe CD649.2. Here, cells after exposure were stained with the CF4 probe at a final concentration of 2 μmol/L for 10 min or CD649.2 at a final concentration of 10 μmol/L for 30 min. The excitation and emission wavelengths were set as mentioned above. Cell organelles lysosomes and mitochondria were stained by 0.5 μmol/L lysosomal tracker (LysoTracker Deep Red DND-99, L7528, Thermo Fisher) and 0.1 μmol/L mitochondrial tracker (MitoTracker Deep Red M22426 or MitoTracker Green M7514, Thermo Fisher) for 1 h and 10 min, respectively. The cellular autophagy was stained with 50 μmol/L monodansyl cadaverine (MDC) (HY-D1027, MCE) for 15 min. After that, cells were washed with 1× PBS twice to remove the residue probes, and the confocal images were captured with a confocal microscope LSM900 equipped with Airyscan in channel mode (Carl Zeiss). For fluorescent signal capture, excitation and emission wavelengths were set as follows: LysoTracker Red: λex = 561/λem = 590–650 nm; MitoTracker Deep Red: λex = 6633/λem = 650–700 nm; MitoTracker Green λex = 488/λem = 500–5550 nm, MDC: λex = 405/λem = 512 nm. Pearson’s correlation coefficient (PCC) was used to quantify the degree of colocalization between Cu(I)/Cu(II) and lysosomes or mitochondria. To analyze the mitochondrial structure, the confocal graphs of mitochondria were treated with the 2D Threshold Optimize function to obtain the optimized Block size and C-value. Then, the treated graphs were analyzed by the 2D Analysis function of this program to derive the parameters of mitochondria, including area, perimeter, aspect ratio, branches number, branch lengths, and branch junctions.

The cellular responses, including ROS formation and GSH formation, were measured via CM-H_2_DCFDA (C6827 Thermo Fisher) and ThiolTracker Violet (T10095 Thermo Fisher). Briefly, SH-SY5Y cells after exposure were incubated with 40 μmol/L CM-H_2_DCFDA regent or 1 μmol/L ThiolTracker Violet for 1 h. The stained cells were then twice washed with 1X PBS to remove the staining solution. The confocal images were captured by the confocal microscope LSM900 (Carl Zeiss) with specific excitation and emission wavelengths (ROS: λex = 488/λem = 525 nm) or (GSH: λex = 405/λem = 525 nm) separately. The mean fluorescence intensity (MFI) of ROS and GSH were quantified by calculating the confocal images. The Cu(I)/Cu(II) were stained and confocal graph were captured as described above. Pearson’s correlation coefficient (PCC) was used to quantify the degree of colocalization between Cu(I)/Cu(II) and GSH.

### Cu Contents in Vivo and in Vitro

The SH-SY5Y cells after exposure were rinsed twice by using 1× PBS and harvested by trypsin and collected by centrifugation at 4 °C, 650*g* for 5 min. The cells were then digested with 200 μL of ultrapure HNO_3_ and heated at 80 °C overnight. For mice samples, about 100 μL of serum from AD or WT mice was mixed using 200 μL of ultrapure HNO_3_ and heated at 80 °C overnight for digestion. About 0.1 g of tail tissue from AD or WT mice was mixed using 200 μL of ultrapure HNO_3_ and heated at 80 °C for 2 days for digestion. Subsequently, the samples were diluted with 2% HNO_3_ before the detection of concentrations of phosphorus and Cu at the same time by using ICP-MS (NexION 300×, PerkinElmer, USA). Phosphorus contents were employed to normalize the cell number. The calibration curve of the phosphorus concentration and cell number was established to quantify the number of cells.

### RNA-Seq Analysis

RNA-Seq raw data (PRJNA728528) were downloaded from the Sequence Read Archive (SRA) (www.ncbi.nlm.nih.gov/geo) in Fstaq format. The data included three ovarian tumors and three normal samples. Ubuntu 17.10 (64-bit) was used to process the raw data and software R (version 3.5.1, https://www.r-project.org/) was used for statistical calculation and interpretation of DEGs. Biological significance of DEGs was explored by GO term enrichment analysis including biological process (BP), cellular component (CC), and molecular function (MF), based on Bioconductor packages enrichR (https://cran.r-project.org/package=enrichR), and then KEGG pathway enrichment analysis of DEGs was performed with enrich R as well.

All of the data were analyzed by SPSS software. One-way *t*-test was used to analyze the difference between control group and each experimental group (*p* < 0.05).

## Results and Discussion

### Toxicity of Aβ1–42 Fibrils

The benzathiole dye Thioflavin-T (ThT) is commonly applied to determine the aggregation statue of amyloid fibrils,^50^ and its assay was conducted each day to follow the fibrosis progression of Aβ. Aβ1–42 fibrils were formed after 3 days and increased with increasing incubation time to 6 days (Figure S2A). The Aβ fibrils were further observed via SEM (Figure S2B), which showed the dot form (monomer) of Aβ at the beginning of incubation, and fibrils were found after 6 days. These results indicated that Aβ formed fibrils after 6 days of incubation and thus could be used in the following study as Aβ aggregates. The aggregation process was consistent with the previous study, in which long and straight Aβ fibrils was formed on day 6 of culture.^51^ To determine the Aβ induced cell death, SH-SY5Y cells were cultured with aggregated Aβ or nonaggregated Aβ at different concentrations (ranging from 1 to 10 μmol/L) for 12 and 24 h. The exposure of Aβ changes the morphology of the SH-SY5Y cell. The arrows indicate the shorter neurite outgrowth of SH-SY5Y cells after Aβ exposure (Figure S3A). Decreased cell viability in a dose-dependent manner was found in the cultured SH-SY5Y cells (Figure S3B). Significant cell death was observed at >5 μmol/L when compared with the nonaggregated Aβ at the same concentration (Figure S3C), demonstrating that only Aβ fibrils caused cytotoxicity, consistent with other neuro cells including BV2 and PC12 cells.^52^ Thus, Aβ fibrils incubated for 6 days at a final concentration of 1 or 5 μmol/L were chosen for low and high exposure dosages in subsequent experiments.

### Changes in Cu Ion Valences under Aβ Addition

To investigate the influence of Aβ addition or AD on Cu ion valences in neural systems, we applied both *in vivo* and *in vitro* models. The contents of Cu *in vivo* and *in vitro* were quantified via ICP-MS (Figure S4). Intracellular Cu content under Aβ exposure did not show a significant change. The Cu content in serum from AD mouse was slightly higher than that from the WT mouse, and other tissue (tail) did not show changes in Cu content. Therefore, Aβ exposure did not alter the total content of Cu in cells, suggesting that Aβ addition had no effect on Cu uptake or release.

**Figure 1:**
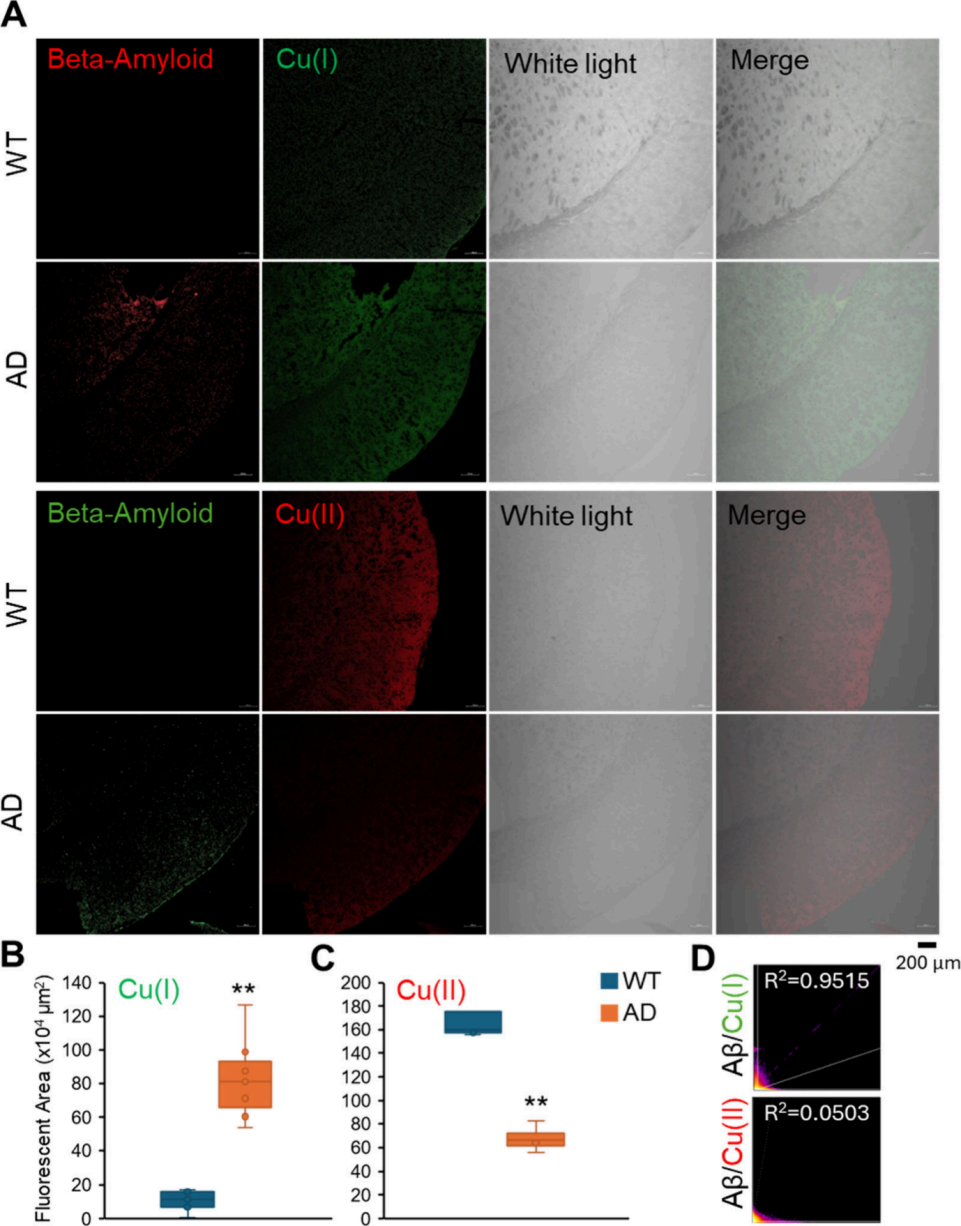
Confocal images of mouse brain tissue section. (A) Brain tissue sections from AD and wild type (WT) mouse were stained with anti-Aβ (red) and Cu(I) (green) (upper panel) and anti-Aβ (green) and Cu(II) (red) (lower panel). The fluorescence intensity of confocal graphs is expressed as the area fluorescent signal of Cu(I) (B) and Cu(II) (C) in each graph. (D) Scatter plot graph of Aβ and Cu(I) (upper panel) and Cu(II) (lower panel) and the value of colocalization ratio (*R*^*2*^). Mean ± SD (*n* = 6). *p < 0.05; **p < 0.01.

For the *in vivo* model, the brains of an AD model mouse and wildtype (WT) mouse were stained with Cu(I) and Cu(II) probes, and Aβ in brain sections was recognized by anti-Aβ antibody whose signal did not show in the WT mouse samples (Figure 1A). The amounts of Cu(I) and Cu(II) were quantified based on the confocal images (Figure 1B and C). Cu(I) increased significantly in AD mouse and was about 8 times higher than that in the WT one. In contrast, Cu(II) decreased by about 3 times under AD conditions. To further prove the relationship between Aβ and changes in Cu ion valences, the colonization ratios between Aβ and Cu(I) and Cu(II) were calculated. The signal of Cu(I) was highly colocalized with Aβ, with a colocalization ratio of ∼0.9 (Figure 1D upper panel). The signal of Cu(II) was not colocalized with Aβ, with a colocalization ratio of ∼0.05 (Figure 1D lower panel). The staining of tissue sections indicated that the Cu valence in the brain of the AD mouse was altered, in which Cu(I) increased, while Cu(II) decreased due to the existence of Aβ.

**Figure 2:**
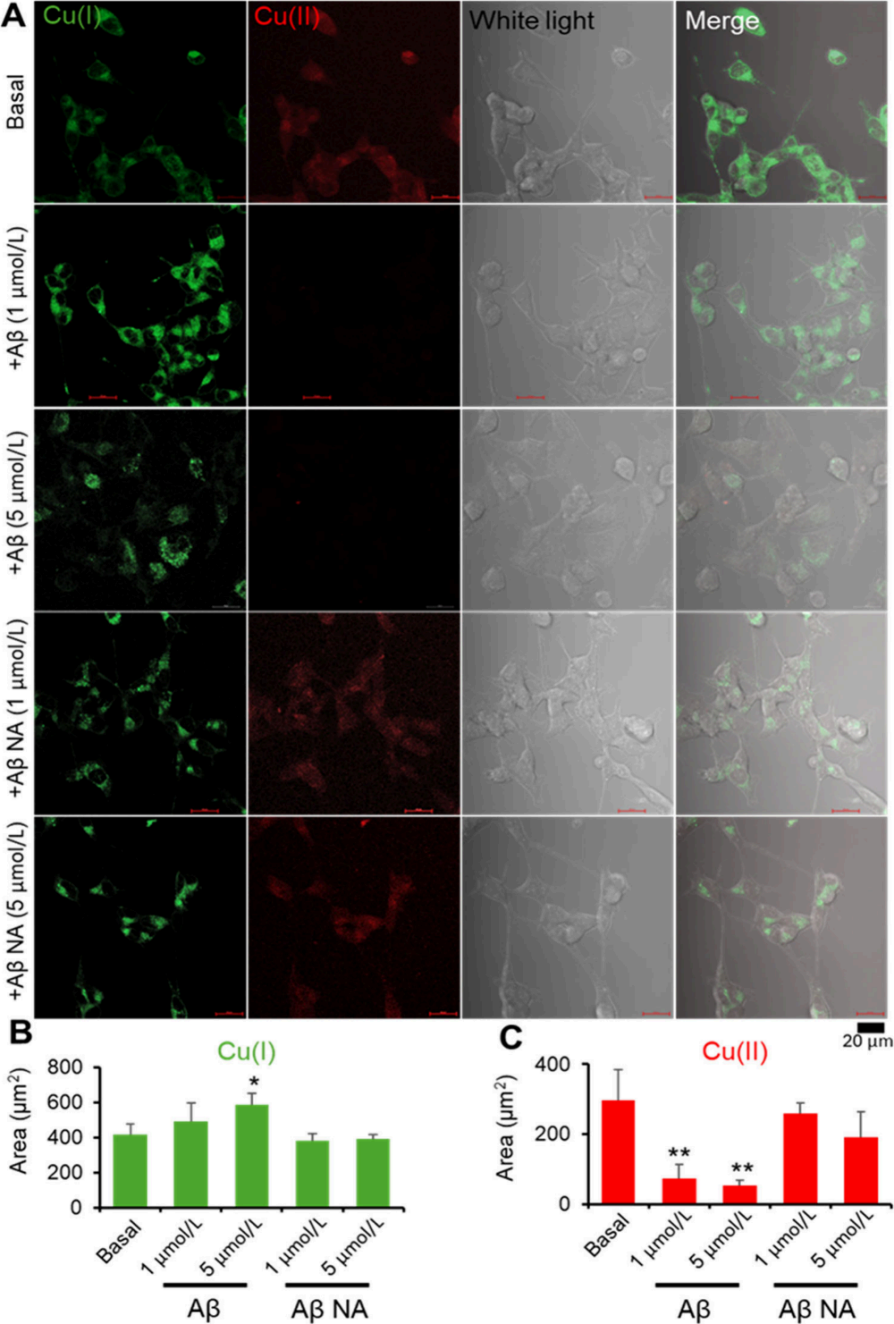
Confocal images of SH-SY5Y under Aβ exposure. (A) Confocal images of Cu(I) (green) and Cu(II) (red) in SH-SY5Y cells after Aβ (+Aβ) and nonaggregate Aβ (+Aβ NA). The fluorescent intensity of confocal graphs is expressed as the area fluorescent signal of Cu(I) (B) and Cu(II) (C) in each graph. Mean ± SD (*n* = 6). *p < 0.05; **p < 0.01.

To further demonstrate the Aβ induced disruption in Cu homeostasis, SH-SY5Y cells were used as an *in vitro* model to reveal the amounts of cellular Cu(I) and Cu(II) under Aβ addition. To confirm the specificity and stability of Cu probes, Z-stack staining images of SH-SY5Y cells without treatment are shown in Figure S5. This confocal graph indicates that these Cu specific probes stained the intracellular Cu (I and II) and cell organelles at the same time. SH-SY5Y cells were stained with Cu(I) and Cu(II) probes after exposure at different concentrations (1 and 5 μmol/L) of Aβ and nonaggregated Aβ for 24 h. The confocal graphs with different treatments are shown in Figure 2A. Under Aβ addition, the fluorescent signal of Cu(I) increased, while the signal of Cu(II) decreased. Meanwhile the nonaggregated Aβ did not cause such changes in Cu valence. The area of Cu(I) and Cu(II) fluorescent signals was calculated based on the confocal graphs, and the quantification results showed that the addition of Aβ caused a dose-dependent increase of cellular Cu(I) amount (Figure 2B) and decrease of Cu(II) amount (Figure 2C). Meanwhile, both low and high dosages of nonaggregated Aβ did not contribute to changes in intracellular Cu(I) and Cu(II) amounts. Based on the *in vivo* and *in vitro* results, intracellular Cu(II) was transferred into Cu(I) under the exposure of aggregated Aβ fibrils or plaques. Therefore, Cu homeostasis was disturbed by Aβ and the break of Cu homeostasis caused cell damage and cytotoxicity afterward. Noticeably, the changes in Cu level were slightly different between cell and tissue section. In the brain tissue section, Cu(I) increased by about 5 times and Cu(II) decreased by about 2.5 times, while in the cell, Cu(I) increased by about 1.5 times and Cu(II) decreased by about 3 times. Such a difference may be due to the different dosages of Aβ, which lead to different reactions of Cu levels.

### Location of Intracellular Cu under Different Conditions

**Figure 3:**
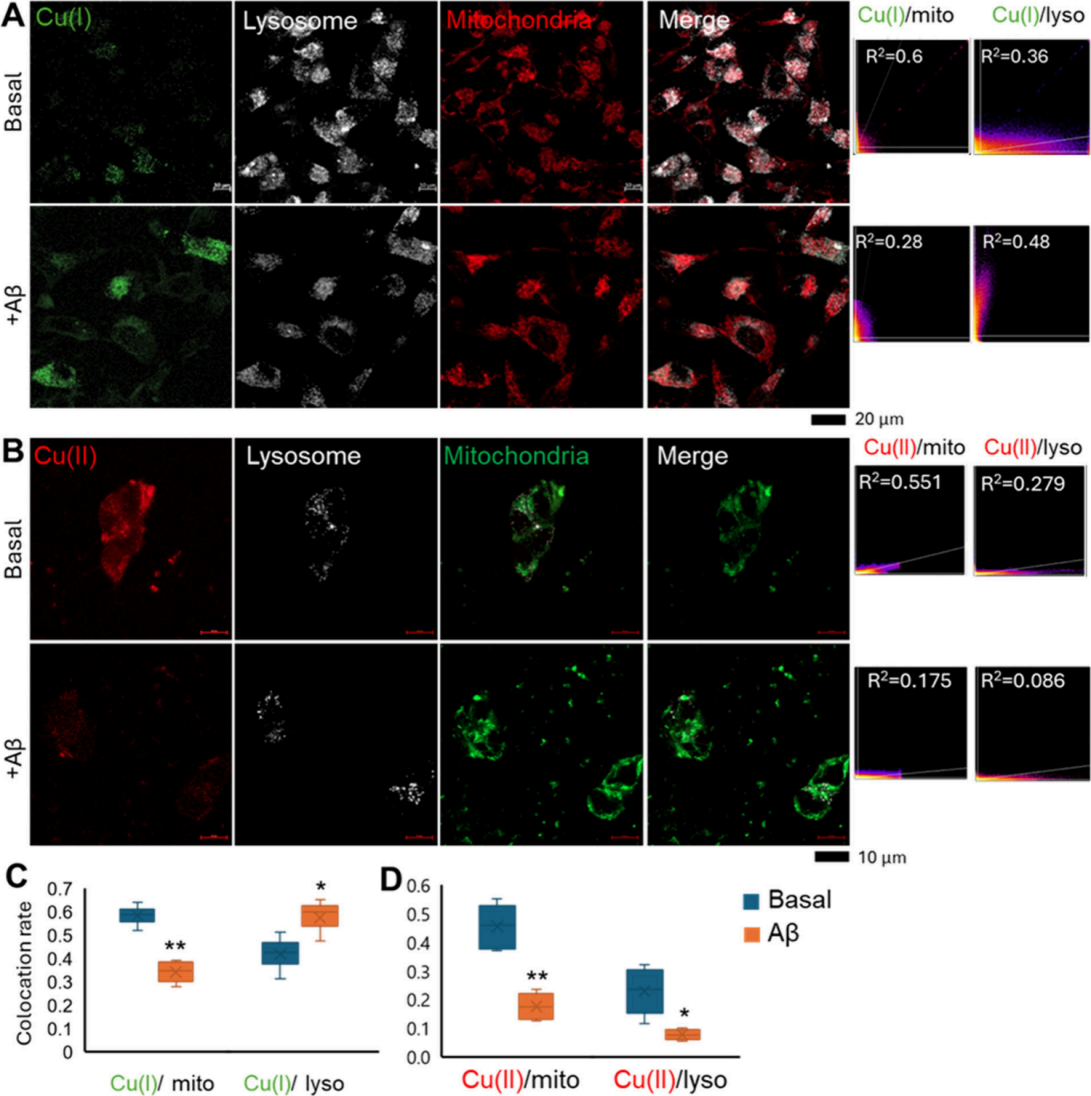
Colocation of Cu(I) and Cu(II) with cellular organelles. (A,B) Confocal images of SH-SY5Y cells after Aβ (+Aβ) exposure. Cellular lysosomes (white), mitochondria (red or green), and Cu(I) (green) and Cu(II) (red). Scatter plot graph of fluorescent signal between mitochondria and lysosome with Cu(I) or Cu(II) (right panel). The value of colocalization ratio (*R*^2^) of the represented graph is shown as well. The colocalization ratio between mitochondria and lysosome with Cu(I) (C) or Cu(II) (D) was calculated based on confocal graphs. Mean ± SD (*n* = 6). *p < 0.05; **p < 0.01.

Lysosomes and mitochondria played critical roles in maintaining Cu homeostasis and detoxification.^53^ To reveal the distribution of Cu *in vitro*, lysosomes and mitochondria of SH-SY5Y cells were stained to visualize their colocalization with Cu(I) and Cu(II) under aggregated Aβ exposure. The confocal images of SH-SY5Y cells at 5 μmol/L Aβ exposure for 24 h are shown in Figure 3A and B.

**Figure 4:**
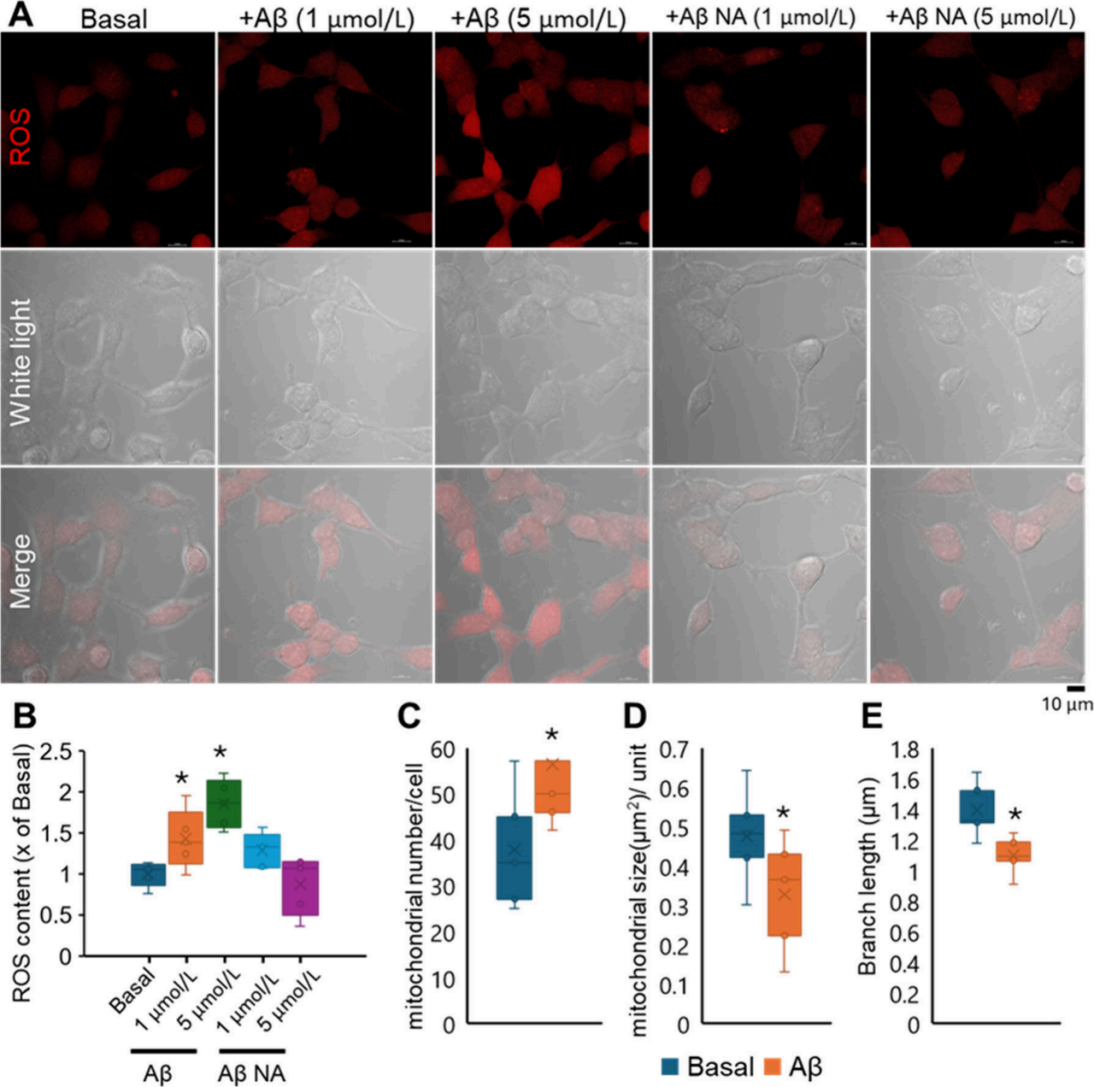
Production of ROS and quantification of mitochondrial morphological changes. (A) Confocal images of SH-SY5Y cells after Aβ (+Aβ) and nonaggregate Aβ (+Aβ NA) exposure and staining with ROS (red). (B) Formation of ROS was quantified via ImageJ. The values are expressed as fold change, as compared with basal reading (as 1, no Aβ added) in mean ± SD (*n* = 6). *p < 0.05. The changes in the mitochondrial network were quantified, and the morphological changes included mitochondrial number (C), mitochondrial size (D), and mitochondrial branch length (E). Mean ± SD (*n* = 6). *p < 0.05.

The colocalization coefficients between mitochondria and lysosomes and Cu(I) (Figure 3C) and Cu(II) (Figure 3D) were measured separately. The scatter plot of fluorescent intensity of three singles are displayed next to confocal graph (Figure 3A and B right panel). Without Aβ addition, Cu(I) was mostly located in the mitochondria (*R*^2^ ∼ 0.6) and only a few Cu(I) was found in the lysosomes (*R*^2^ ∼ 0.3) (Figure 3C). Under Aβ exposure, the colocalization coefficient of Cu(I) with mitochondria decreased significantly (Figure 4C, about 0.3), and more Cu(I) was transferred into lysosomes, which increased from 0.3 to about 0.5. This result indicated that Cu(I) formed under Aβ exposure was mostly located in lysosomes. Meanwhile, the colocalization coefficient of Cu(II) with these two organelles both decreased significantly under Aβ exposure, indicating that Aβ facilitated the transfer of Cu(II) in these organelles to Cu(I). Hence, we hypothesized that Aβ could reduce Cu(II) into Cu(I) in both mitochondria and lysosomes, causing excessive Cu(I) accumulation in mitochondria. Cu(I) was then further transported to lysosomes as a process of detoxification, consistent with earlier findings.^53^ Based on these findings, we hypothesized that the cytotoxicity of Aβ might originate from the Cu(I) that was transferred from Cu(II), and the excessive Cu(I) contributed to the mitochondrial damage and dysfunction this organelle.^54^ Thus, the cellular responses were further investigated to test our hypothesis.

### Cellular Responses under Aβ Addition

Earlier studies demonstrated the increased production of reactive oxygen species^18^ in AD patients and in AD transgenic mouse models.^55^ In our study, intracellular ROS (Figure 4A) increased significantly under Aβ addition in a dose-dependent manner (Figure 4C). In contrast, nonaggregated Aβ addition did not enhance the ROS production. Excessive ROS formation was another factor that led to neurotoxicity under Aβ exposure. However, overproduction of ROS was also related to excessive Cu^56^ or Cu-Aβ complex.^57^ Aβ has been proved to create a microenvironment that facilitates the electron transfer from Cu(II) to O_2_ and generate the peroxide anion (O_2_^2–^) or (O_2_^–^), which subsequently undergoes dismutation to H_2_O_2_.^37^ Therefore, the internal process of ROS over production remains to be investigated.

Mitochondria host the important tricarboxylic acid cycle, oxidative phosphorylation, and ATP production, and control cell differentiation and death, as well as immunological response.^58^ This organelle is an important source of ROS, and excessive Cu(I) in this organelle could cause toxicity.^59^ Changes in mitochondria were investigated to further reveal the cellular response to Aβ addition. Based on the graphical analysis, mitochondrial number, size, and network were measured (Figure 4C–E). Mitochondrial number increased significantly, whereas their sizes decreased significantly from about 0.5 to 0.3 μm^2^ (Figure 4C,D), which may be associated with the break of mitochondrion into several smaller units. Additionally, the mitochondrial network was altered, with a reduced branch and length of mitochondria (Figure 4E). The changes in mitochondrial morphology were in line with the human astrocytes under Aβ accumulation,^60^ as well as the finding that Aβ accumulation caused mitochondrial dysfunction.^61^ Additionally, the shrink of mitochondrial indicated that the dysfunction of mitochondria may come from excessive Cu(I) in this organelle.^62^

**Figure 5:**
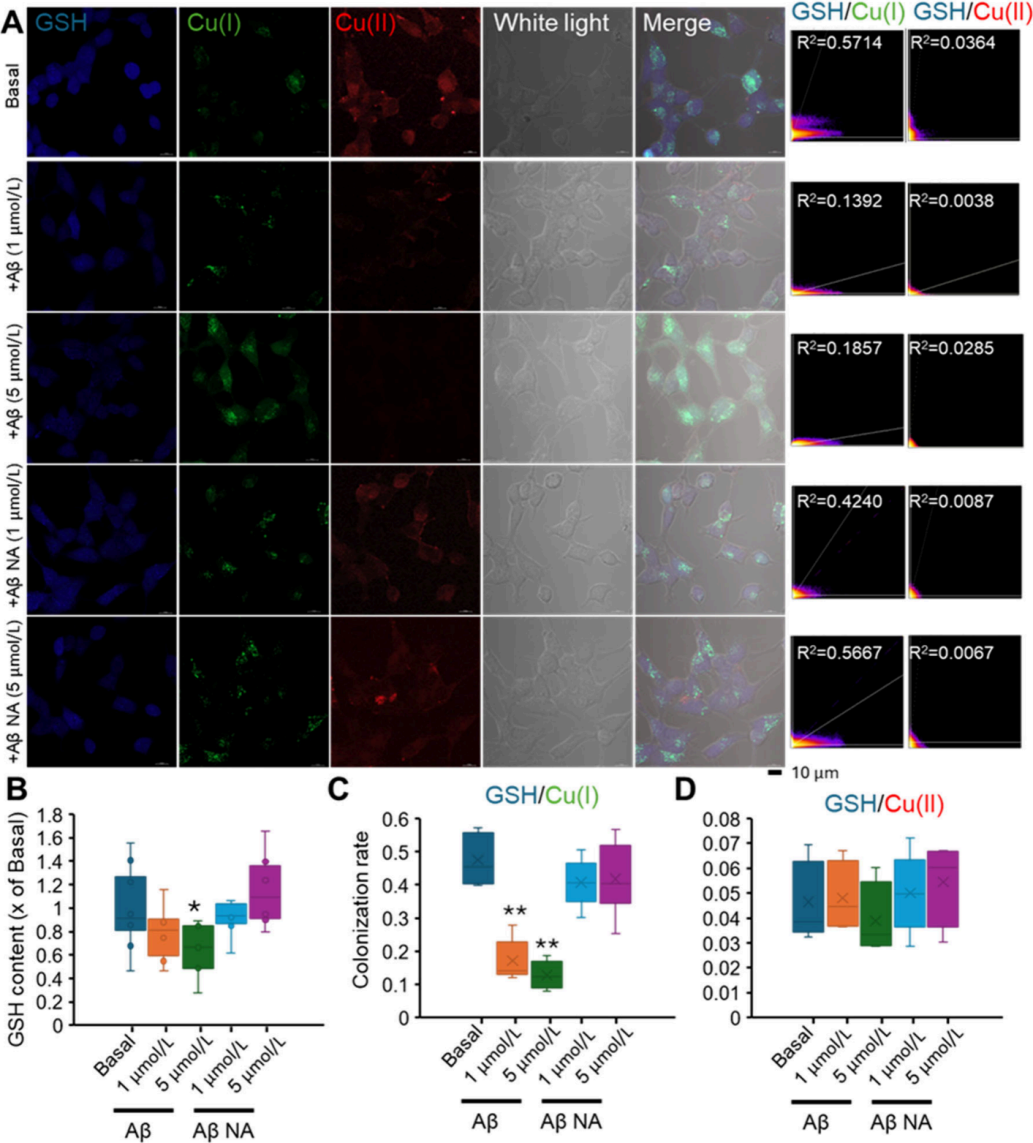
GSH content and colocalization ratio between GSH with Cu(I) and Cu(II). (A) Confocal images of SH-SY5Y cells after Aβ (+Aβ) and nonaggregate Aβ (+Aβ NA) exposure and stained with GSH (blue), Cu(I) (green), and Cu(II) (red). The scatter plot graph of fluorescent signal between GSH with Cu(I) or Cu(II) (right panel) and the value of colocalization ratio (*R*^2^) of represented graph are shown. (B) Formation of GSH was quantified via ImageJ, expressed as fold change as compared with basal reading (as 1, no Aβ added) in mean ± SD (*n* = 6). *p < 0.05. (C) Colocalization ratio between GSH with Cu(I) (C) and Cu(II) (D) under different exposure conditions. Mean ± SD (*n* = 6). *p < 0.05.

Glutathione is a ubiquitous thiol-containing tripeptide containing l-cysteine, l-glutamic acid, and glycine, and one of the most important antioxidants in cells.^63−65^ The balance between ROS and GSH is critical for the maintenance of normal cellular functions.^19^ To investigate the cellular response under Aβ exposure, the intracellular GSH was quantified as well (Figure 5A,B). Here, the GSH content decreased significantly under high Aβ exposure, whereas nonaggregated Aβ addition did not alter the GSH content in cell at all dosages. The decreased GSH might be due to the increasing GSSG (oxidized form) under oxidative stress with excessive ROS. This result was different from previous study in which GSH level did not alter under Aβ exposure,^66^ possibly due to difference in exposure period and cell type.

Apart from participating in detoxification of reactive species and electrophiles, GSH also binds with intracellular metal ions.^67^ Cu(I) in cytoplasm was mostly localized with the GSH,^53^ and the decrease of GSH might release Cu(I) into the cytoplasm. To confirm this hypothesis, the amounts of Cu(I) and Cu(II) bound with GSH were also quantified (Figure 5A). The scatter plots of fluorescent intensity of three singles are shown next to the confocal graph (Figure 5A right panel). Half of Cu(I) was bound with GSH (*R*^2^ ∼ 0.6) without Aβ addition, whereas the colocalization coefficient of Cu(I) with GSH decreased significantly (Figure 5B) to about 0.2 when Aβ was added. Increasing Aβ concentration resulted in a further loss of Cu(I) bound with GSH, but the nonaggregated Aβ did not cause changes in colocalization coefficients of GSH and Cu(I). This result indicated that Aβ addition resulted in a release of Cu(I) from GSH into cytoplasm. Meanwhile, almost no Cu(II) was bound with GSH (*R*^2^ ∼ 0.03) under all exposure conditions.

Based on the amyloid cascade hypothesis, many brain imaging tools including magnetic resonance imaging (MRI), computerized tomography,^2^ and positron emission tomography (PET) are applied in the clinical diagnosis of AD^68^ through brain imaging of Aβ fibrils. However, with the imaging technology limitation, the detection of Aβ fibrils usually representes late stage of AD, thus considerably delaying the diagnosis. Based on our results, the changes in the Cu(I) and Cu(II) ratio were significant under a very low dosage of Aβ fibrils exposure. Therefore, fluorescent Cu(I) and Cu(II) imaging may potentially provide a sensitive early potential marker in AD’s diagnosis.

### Transcription Analysis under Aβ Addition

**Figure 6:**
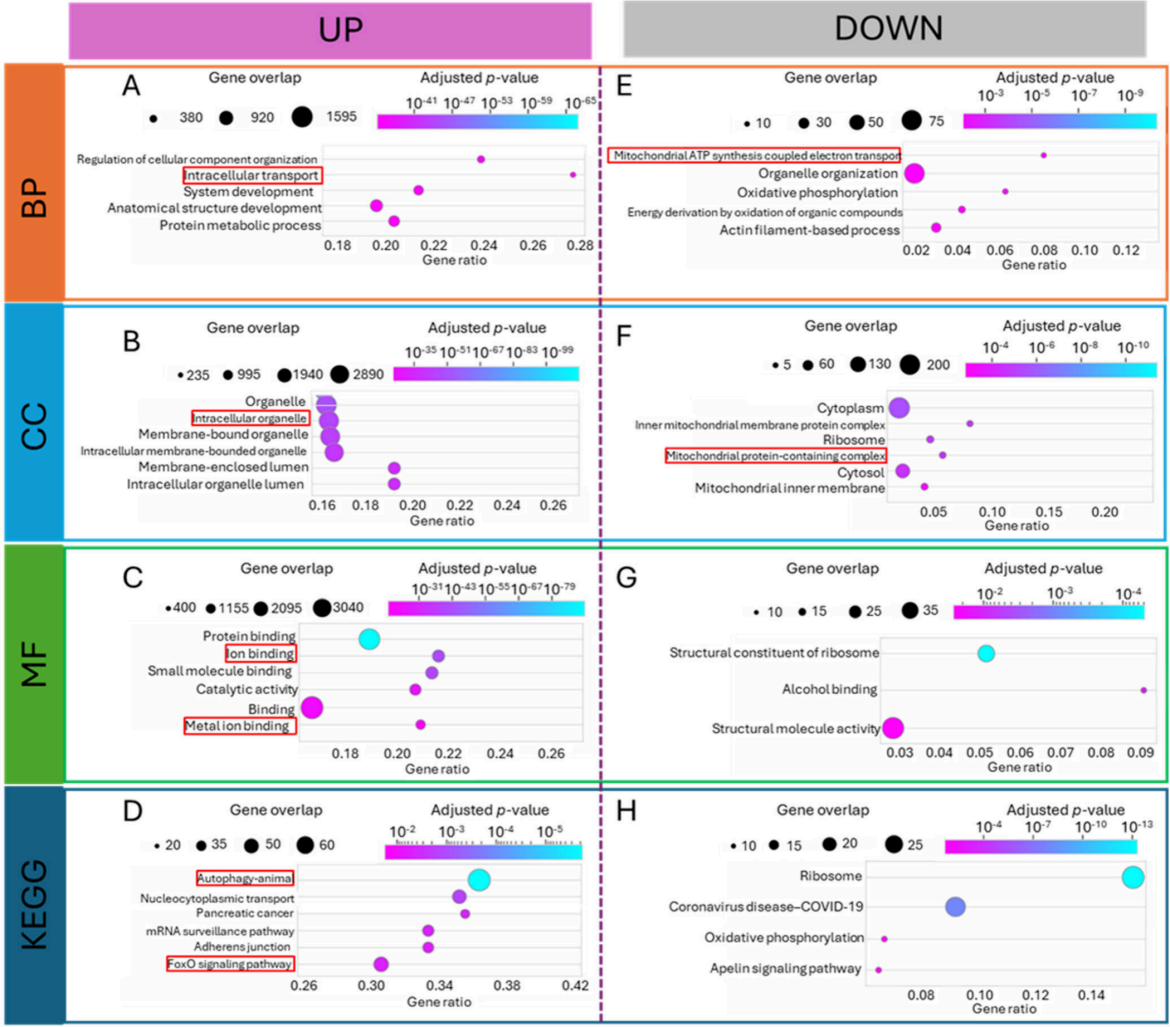
Functional enrichment analysis under Aβ exposure. (A–C) GO function enrichment analysis of upregulated DEGs. (D) KEGG pathway enrichment analysis of upregulated DEGs. (E–G) GO function enrichment analysis of downregulated DEGs. (H) KEGG pathway enrichment analysis of downregulated DEGs.

In order to further reveal the mechanism of Aβ caused disruption of Cu homeostasis and cellular responses, transcription analysis was performed by using RNA sequencing data from previous research.^63^ SH-SY5Y cells after Aβ1–42 was added at the final concentrations of 4 μmol/L,^63^ and the exposure conditions were similar to the present study. When the AD model was compared with control cells, 4139 differentially expressed genes (DEGs) were identified as having significant changes by using a fold change cutoff ratio of ≥2 or ≤0.5 (3721 upregulated, 418 downregulated). We further analyzed the cellular functions through the GO (gene ontology) database and KEGG (Kyoto Encyclopedia of Genes and Genomes) enrichment. Here, biological process (BP), cellular component (CC), and molecular function (MF) of upregulated and downregulated genes are shown in Figure 6. The box with the same color represents the gene functions enriched in certain databases (BP, CC, or MF). The up-regulated and down-regulated genes are shown in the right or left panel of each box. Among the most 20 abundant enriched gene functions, the functions with potential relationship with Cu, Cu transportation (intracellular transportation), and autophagy were picked manually and marked with red frames based on the literature review. Overall, genes related to intracellular transport (Figure 6A) and intracellular organelles (Figure 6B) were upregulated under Aβ exposure, suggesting that the increase of subcellular transportation especially the interactions between cellular organelles. Such changes were in line with previous research that genes related to intracellular transport^69^ and intracellular organelles^70^ were highly associated with Cu homeostasis. Also, these increased cellular functions were also found in Cu-induced autophagy.^71^ Additionally, increased metal ion binding was found in molecular function (Figure 6C), in line with the cells under Cu exposure.^72^ Meanwhile, gene functions related with mitochondrial ATP synthesis coupled electron transport and mitochondrial protein-containing complexes were found in downregulated genes. These responses were similar to the excessive copper in cells.^73,74^ These cellular functions increased by Aβ exposure were partly similar to Cu exposure which represented the existence of excessive Cu in cells.

For the KEGG result, autophagy was the most enriched pathway that was upregulated, similar to cells under Cu exposure.^75^ Meanwhile, the signal pathway FoxO (Forkhead box O) was upregulated, similar to Cu exposure in human cells.^76^ Overall, the similar upregulated gene pathways under Aβ exposure as those under Cu exposure further suggested that Aβ exposure caused excessive Cu(I) in cells. To prove the transcription result, the autophagy in cells was stained and quantified (Figure S6A). The Aβ exposure increased the cell autophagy about 4 times compared with the basal or nonaggregated Aβ (Figure S6B). Additionally, genes related to mitochondrial ATP synthesis (Figure 6E) and mitochondrial protein-containing complexes (Figure 6F) were downregulated, indicating damage in mitochondria. These transcription results were similar to those of TNBC cells under excessive Cu exposure,^77^ which represented the damage of mitochondrial caused by Cu. The transcription analysis supported our hypothesis that Aβ disrupted Cu homeostasis and excessive Cu(I) contributed to the damage of mitochondria. By combining the transcription analysis and autophagy staining, excessive Cu(I) caused by Aβ exposure could lead to mitochondrial damage, cell death, and autophagy afterward. The combination of transcription analysis and staining further proved that the distribution of copper homeostasis could induce autophagy in cells under Aβ exposure.

Apart from functional analysis, the expression levels of APT7A and APT7B were found to increase by about 3.5- and 2.0-fold under Aβ exposure. These two genes contributed to Cu transportation.^78^ The increasing APT7A expression indicated the transportation of Cu ion from cytoplasm into lysosome.^79^ This finding further proved our hypothesis that Aβ exposure resulted in a reduction of Cu(II) to Cu(I) and excessive Cu(I) was further transported into the lysosome to detoxify the overloaded Cu.

This study revealed the disruption of Cu homeostasis in AD mice and cells under Aβ addition. With the bioimaging tools, the distribution and valence of intracellular Cu(I) and Cu(II) under Aβ exposure was investigated. In both *in vitro* and *in vivo* models, more Cu(I) but fewer Cu(II) was found, indicating that Cu(II) was reduced to Cu(I), while nonaggregate Aβ did not cause changes in Cu valence. In the cell model, excessive Cu(I) triggered by Aβ was mainly found in the mitochondria, and lysosomes played a detoxification role by mitophagy. Meanwhile, excessive Cu(I) led to over production of ROS and changes in mitochondrial morphology, suggesting the damage of Cu(I) and dysfunction of mitochondria. With excessive ROS, GSH in the cytoplasm was oxidized into GSSG, with a further loss of Cu(I) and accumulation in the cytoplasm as well as lysosome. RNA sequencing analysis showed that genes related to intracellular transport, intracellular organelle, and metal ion binding increased, whereas the down-regulated genes represented the mitochondrial damage. In particular, the expression of ATP7A/B as the two Cu ion transporters increased by 3 times, indicating the role of lysosome in detoxification of Cu(I) in Aβ exposure. These changes in transcription were found to be in line with excessive Cu exposure, indicating that the damage was caused by Cu. This study demonstrated that Aβ exposure caused the disruption of intracellular homeostasis by reducing Cu(II) to Cu(I) and damaging the mitochondria, which further triggered the detoxification by lysosome. Our finding provided new insights into Aβ and AD induced Cu redox transformation and toxicity. Thus, these findings revealed another factor causing AD, which may provide potential markers in diagnosis and therapy.

## Conclusion

Previous studies primarily focused on the Cu amounts in AD patients or the Aβ-Cu complex reaction in an abiotic environment. In this work, the change in Cu valences was visualized both in cells and the mouse AD model using specific fluorescent probes. According to the image analysis, Cu(I) was found to increase, while Cu(II) decreased under AD conditions. Both *in vivo* and *in vitro* results showed that the Cu(I)/Cu(II) ratio was strongly related to the existence of Aβ. Imaging and transcription analysis further revealed that Aβ and AD induced Cu redox transformation, which caused the disruption of intracellular Cu homeostasis and damaged the mitochondria. This study revealed that changes in the Cu(I)/Cu(II) ratio might provide potential markers in the diagnosis and therapy of this disease.

## Supporting Information Available

Brain tissue section of mouse; aggregation process of Aβ fibrils; cytotoxicity of aggregated and nonaggregated Aβ fibrils SH-SY5Y cells; calibration curve of ICP-MS intensity and Cu content and cell number and Cu content in cells and tissue samples; Z-stack image of SH-SY5Y cells after staining; utophagy of SH-SY5Y cells after Aβ addition (PDF)
