# Supplementary material for: Disruption of Copper Redox Balance and Dysfunction under In Vivo and In Vitro Alzheimer’s Disease Models
Source: Environ Health (Wash). 2024 Nov 13;3(3):238–49. doi: 10.1021/envhealth.4c00175 (PMC11934196; doi:10.1021/envhealth.4c00175)
Supplement: Supplementary file 1 — eh4c00175_si_001.pdf [file eh4c00175_si_001.pdf]

Supporting Information for

Disruption of Copper Redox Balance and Dysfunction Under in Vivo and in Vitro Alzheimer Disease Models

Yiteng Xia[1,2], Karl W.K. Tsim[3], Wen-Xiong Wang[1,2]*

[1]School of Energy and Environment and State Key Laboratory of Marine Pollution, City University of Hong Kong, Kowloon, Hong Kong, China

[2]Research Centre for the Oceans and Human Health, City University of Hong Kong Shenzhen Research Institute, Shenzhen 518057, China

[3]Division of Life Science, Hong Kong University of Science and Technology, Clear Water Bay, Kowloon, Hong Kong, China

*Corresponding author, Email: wx.wang@cityu.edu.hk

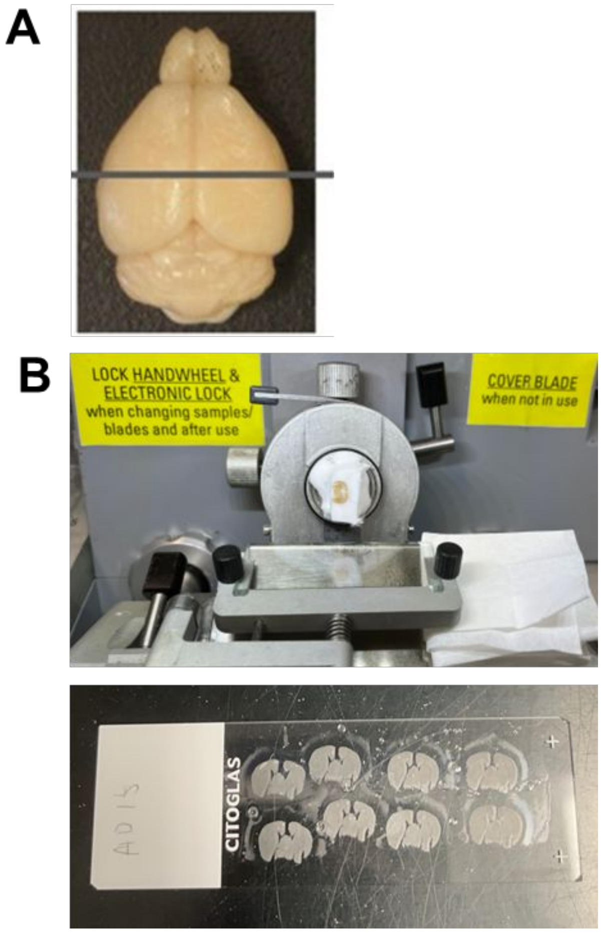

Figure S1. Brain tissue section of mouse. (A) The coronal tissue sections of mouse brain were prepared in this study. (B) The brain tissues before and after the cryosections.

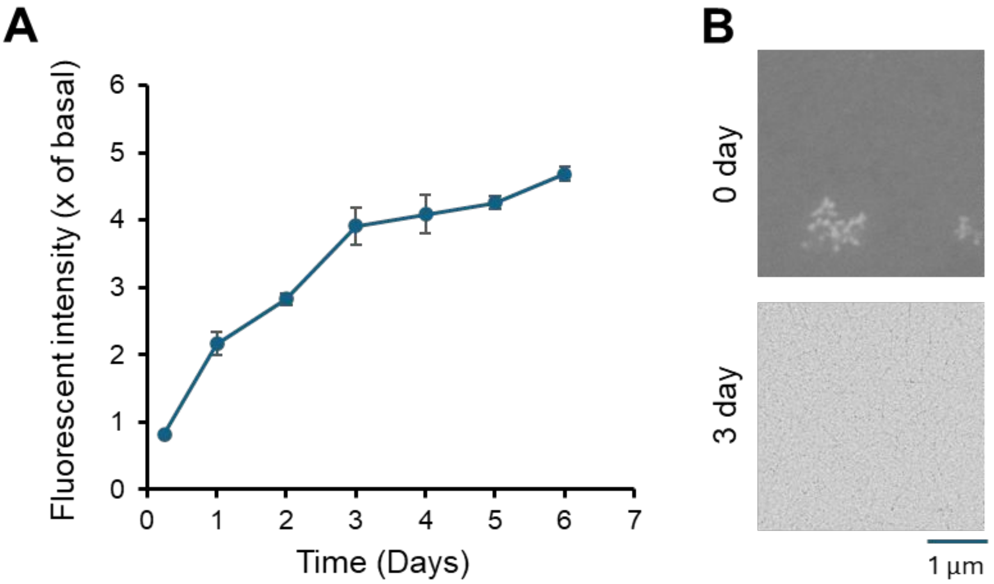

Figure S2. Aggregation process of Aβ fibrils. (A) The fluorescent intensity of (ThT) assay of Aβ fibrils during 6-day incubation. The values are expressed as fold of changes, as compared with basal reading (as 1, at day 0). Data are Mean ± SD (n = 6). (B) The SEM graphs of Aβ1-42 fibrils during the aggregation process.

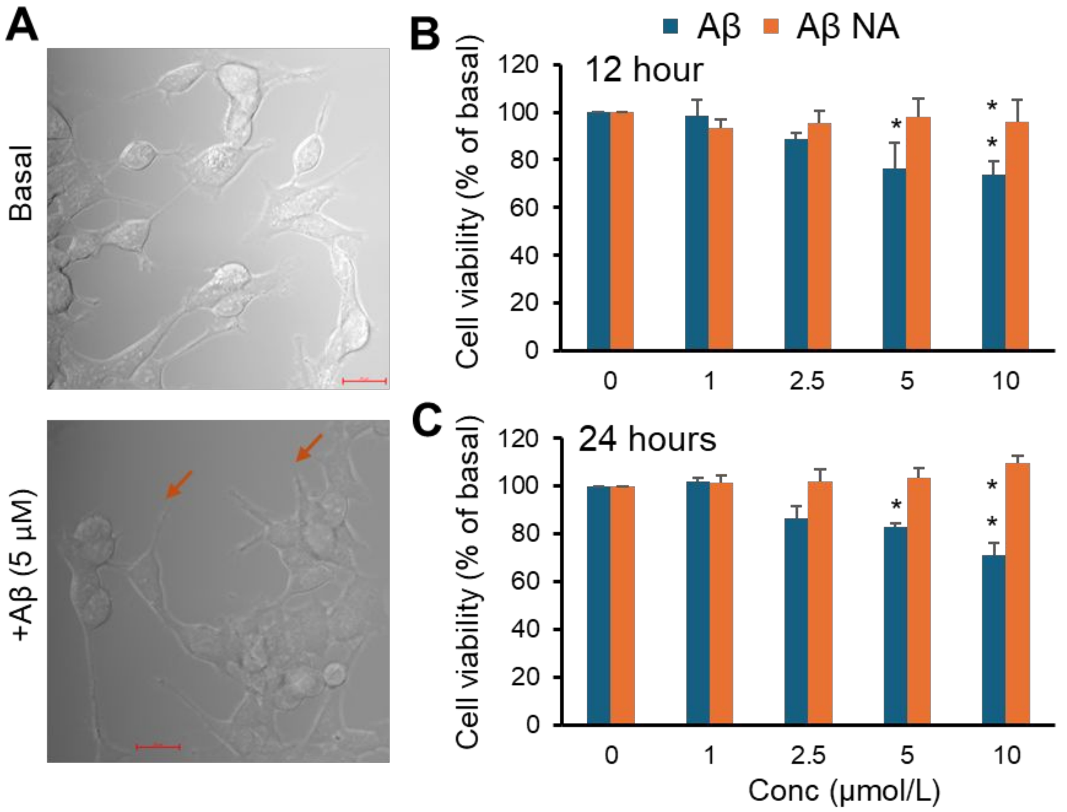

Figure S3. The cytotoxicity of aggregated and non-aggregated Aβ fibrils SH-SY5Y cells. (A) The changes in morphology of cells under Aβ exposure for 24 hours. Arrows point out the shorter neurite outgrowth. Cell viability of the SH-SY5Y cells after exposure to different concentrations of aggregated and non-aggregated Aβ (Aβ NA) fibrils for 12 h **(B)** or 24 h **(C)**. The cell viability was measured via MTT assay. The values are expressed as % of basal, as compared with basal reading (as 100%, no Aβ added). Mean ± SD (n = 6).

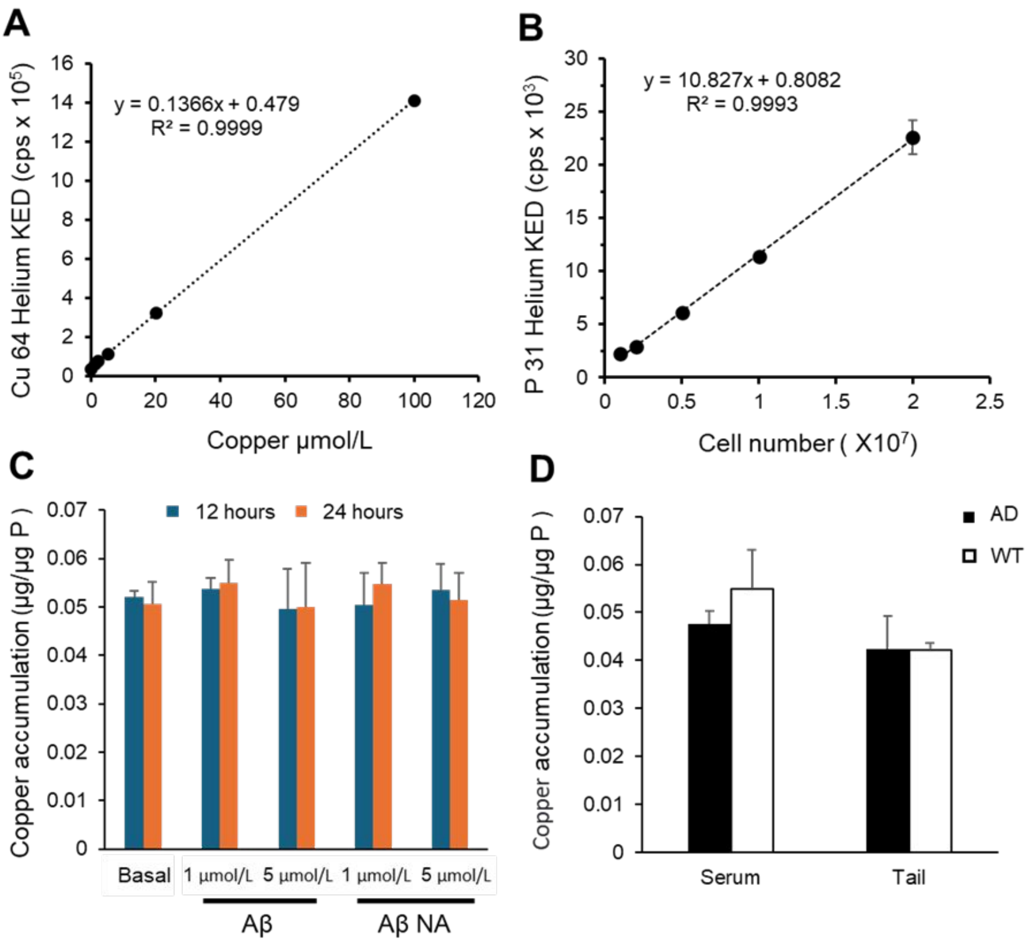

Figure S4. The calibration curve of ICP-MS intensity and Cu content and cell number and Cu content in cells and tissue samples. (A) The CPS (count per second) value of copper ion from ICP-MS was compared with the copper ion concentration added. **(B)** The CPS (count per second) value of phosphorus from ICP-MS was compared with the cell number. The equation of liner phase and R-squared value are shown as well. The copper contents in-vitro and in-vivo were determined via ICP-MS. **(C)** The amount of intracellular copper under different exposure concentrations and times. Mean ± SD (n = 3). **(D)** The amount of copper content in different tissues from wild type (WT) and AD mouse. Mean ± SD (n = 3).

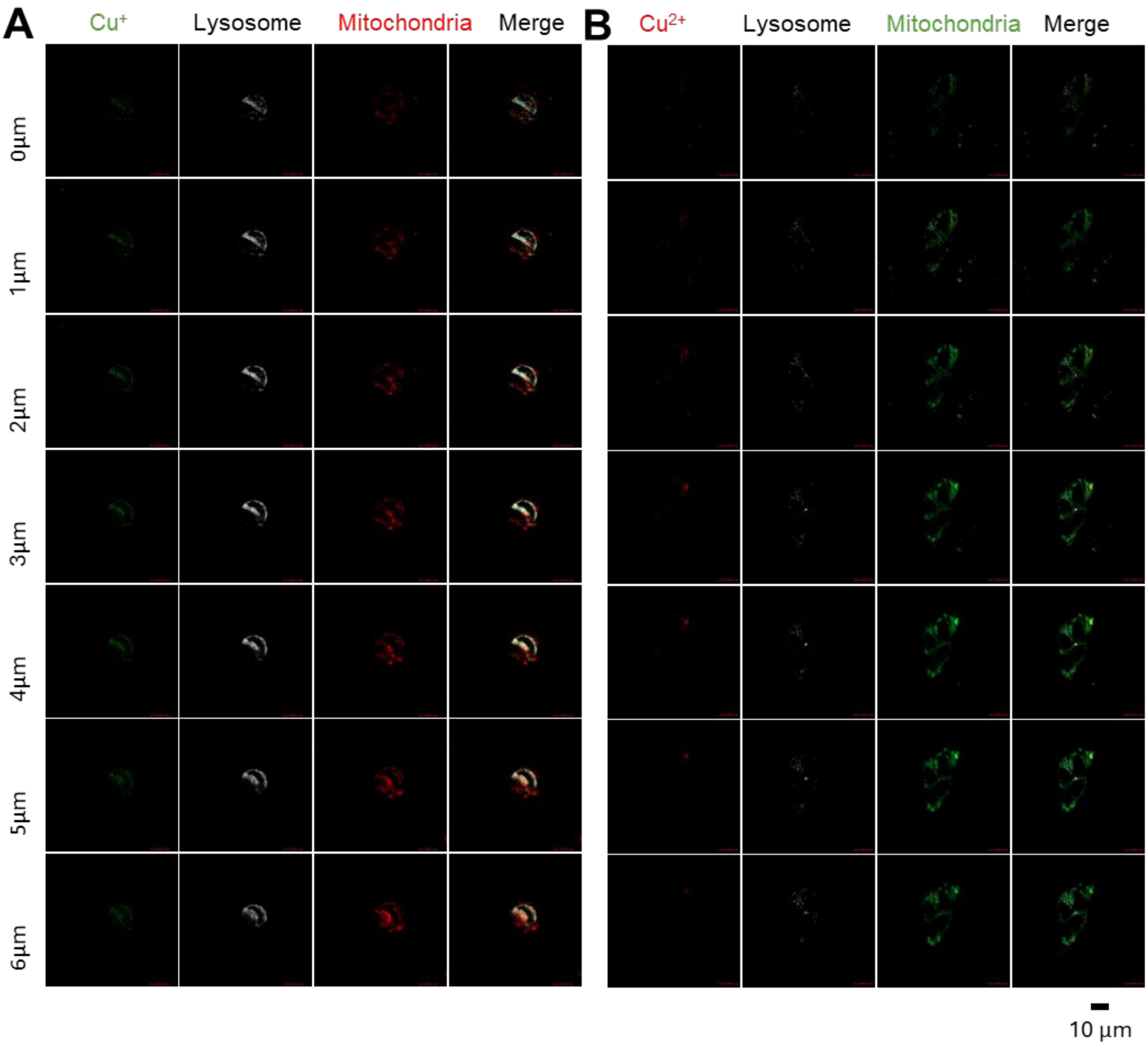

Figure S5. Z-sack image of SH-SY5Y cells after staining. SH-SY5Y cells were seeded on confocal dish for 12 hours, then the intracellular Cu(I) (**A**) and Cu(II) (**B**) and lysosome and mitochondria were stained. The staining process was the same as described in the Materials and Methodst. The images of SH-SY5Y cells at different heights (0~6.0 μm) were taken, and representative photo is shown.

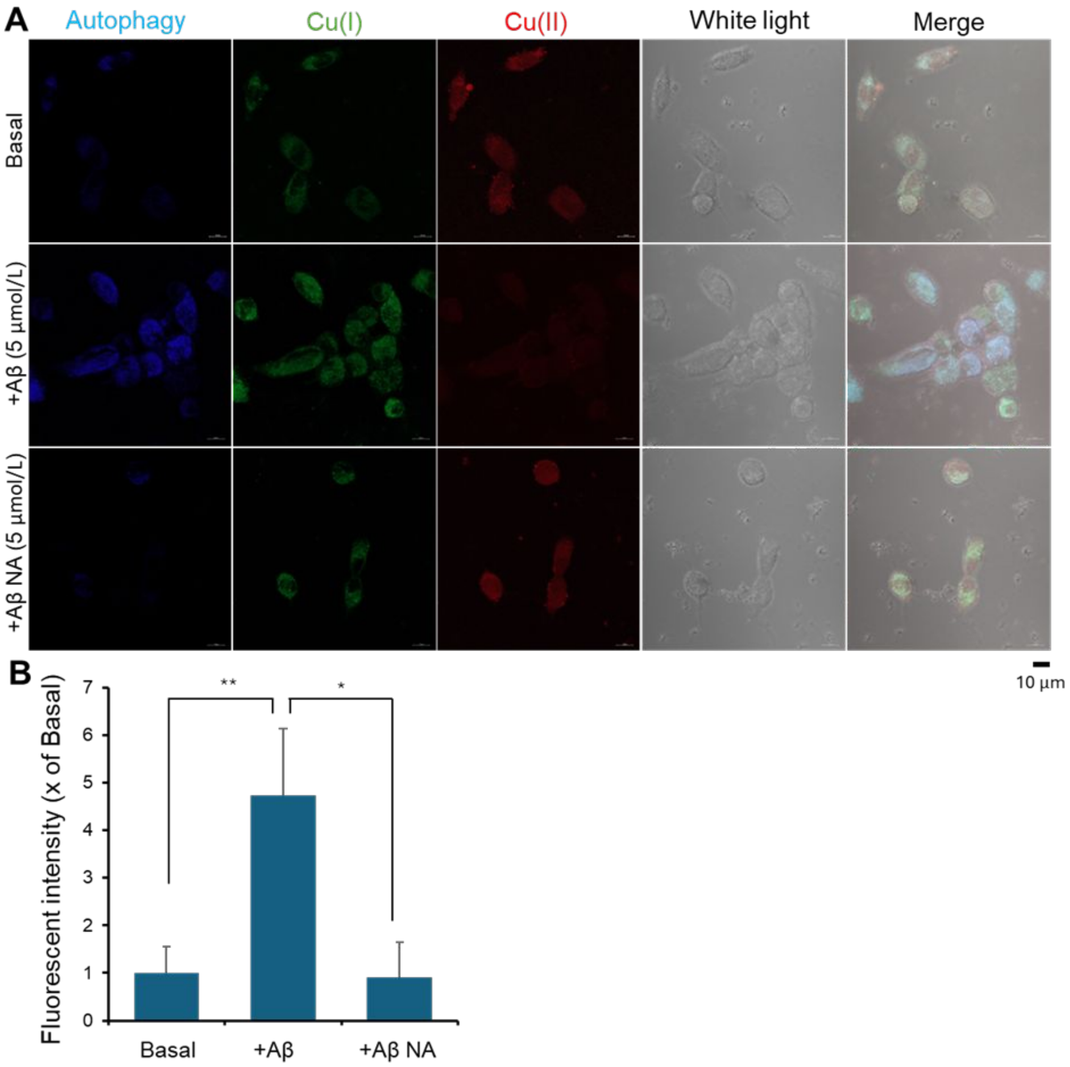

Figure S6. Autophagy staining of SH-SY5Y cells after Aβ. (A) SH-SY5Y cells were seeded on confocal dish for 12 hours, then the autophagy, Cu(I) and Cu(II) were stained. The staining process was the same as described in the Materials and Methods. The images of SH-SY5Y were taken, and representative photo is shown. **(B)** The quantification of fluorescent intensity of MDC (autophagy) in three treatment groups. Mean ± SD (n = 6) * $p < 0.05$, ** $p < 0.05$.
